# Improved Chrysin Production by a Combination of Fermentation Factors and Elicitation from *Chaetomium globosum*

**DOI:** 10.3390/microorganisms11040999

**Published:** 2023-04-12

**Authors:** Siya Kamat, Madhuree Kumari, Kuttuvan Valappil Sajna, Sandeep Kumar Singh, Kaushalendra, Ajay Kumar, C. Jayabaskaran

**Affiliations:** 1 Department of Biochemistry, Indian Institute of Science, Bangalore 560012, India; 2 Celignis Biomass Analysis Laboratory, V94 7Y42 Limerick, Ireland; 3 Division of Microbiology, Indian Agricultural Research Institute, Pusa, New Delhi 110012, India; 4 Department of Zoology, Mizoram University (A Central University), Pachhunga University College Campus, Aizawl 796001, India; 5 Centre of Advanced Study in Botany, Banaras Hindu University, Varanasi 221005, India

**Keywords:** chrysin, flavonoid, elicitors, endophyte, marine fungus, *Chaetomium globosum*, LC-MS/MS

## Abstract

Flavonoids encompass a heterogeneous group of secondary metabolites with exceptional health benefits. Chrysin, a natural dihydroxyflavone, possesses numerous bioactive properties, such as anticancer, antioxidative, antidiabetic, anti-inflammatory, etc. However, using traditional sources of chrysin involves extracting honey from plants, which is non-scalable, unsustainable, and depends on several factors, including geography, climatic conditions, and the season, which limits its production at a larger scale. Recently, microbial production of desirable metabolites has garnered attention due to the cost-effectiveness, easy scale-up, sustainability, and low emission of waste. We previously reported for the first time the chrysin-producing marine endophytic fungus *Chaetomium globosum*, associated with a marine green alga. To extend our understanding of chrysin biosynthesis in *C. globosum,* in the present study, we have assessed the presence of flavonoid pathway intermediates in *C. globosum* extracts using LC-MS/MS. The presence of several key metabolites, such as dihydrokaempferol, chalcone, galangin, baicalein, chrysin, p-Coumaroyl-CoA, and p-Cinnamoyl-CoA, indicates the role of flavonoid biosynthesis machinery in the marine fungus. Further, we have aimed to enhance the production of chrysin with three different strategies: (1) optimizing the fermentation parameters, namely, growth medium, incubation time, pH, and temperature; (2) feeding key flavonoid pathway intermediates, i.e., phenylalanine and cinnamic acid; (3) elicitation with biotic elicitors, such as polysaccharide, yeast extract, and abiotic elicitors that include UV radiation, salinity, and metal stress. The combined effect of the optimized parameters resulted in a 97-fold increase in the chrysin yield, resulting in a fungal cell factory. This work reports the first approach for enhanced production of chrysin and can serve as a template for flavonoid production enhancement using marine endophytic fungi.

## 1. Introduction

The health benefits of flavonoids have galvanized the food supplement and nutraceuticals industry [1]. Chrysin (5,7-dihydroxyflavone) is a flavonoid found in honey, propolis, passion flowers, and mushrooms [2]. Increasing evidence indicates that chrysin is associated with health-promoting properties, such as anticancer [3], antioxidant [4], anti-inflammatory [5], antidiabetic [4], neuroprotective [6], hepatoprotective [7], and cardioprotective [8]. We reported chrysin for the first time as a microbial secondary metabolite from an endophytic fungus, *Chaetomium globosum*, associated with a marine green alga, *Chaetomorpha media* [9] (Supplementary Figure S1).

The health-promoting properties of chrysin have inspired efforts in developing chrysin derivatives [10,11,12], chrysin nanoemulsions [13,14,15,16] and nanocomposites [17,18], which are more potent than the parent compound. Previous studies have reported varying chrysin content in passion flowers, honey, mushrooms, and propolis, attributed to the source material’s geography, ecology, or developmental stage [19,20]. The extraction yield also differs due to the breeding seasons, climate conditions, sample size, and variation in species. Chemical synthesis is a favorable option, but the complexity of the flavonoid carbon skeleton renders the chemical synthesis approach challenging and time-consuming and uses hazardous chemicals [21].

On the other hand, fungi possess biotechnological advantages—they are easy to grow and manipulate for higher product yield and pave the way for a sustainable circular economy [22,23]. Endophytic fungi, a diverse group of microorganisms that live within plants or animals imperceptibly, can synthesize various host-derived or novel metabolites [24,25]. Various viewpoints and strategies are discussed to translate fungi into industrial microbial factories. These include strain improvement, genome shuffling, pathway engineering, combinatorial biosynthesis, and mutasynthesis. Other well-sought methods are optimizing the medium and fermentation conditions, engineering the precursor supply for product improvement, and generating novel analogs [24]. Some examples include a 30-fold enhancement in phenolic content was achieved in *Lactuca indica L*. by methyl jasmonate elicitation and culture medium manipulation [26]; a ~1.7-fold enhancement in flavonoid content was achieved in *Tetrastigma hemsleyanum* by metal elicitation (Cu^2+^, Ce^3+^) [27]; a 1.5-fold increase in total phenolic content was achieved by polysaccharide elicitation in *Hypericum perforatum* [28]; a combination of abiotic stress (water stress) and biotic stress (nematode infection) demonstrated a varied response in tomatoes—low phenolic content when water stressed, high flavonoid content when nematode stressed, and high sugar and chlorogenic acid levels when exposed to both stresses [29]; and young poplar plants exposed to water stress demonstrated a decrease in phenolics [30].

Use of this approach can remarkably shorten the fermentation periods and optimize the use of various inexpensive media, feeding precursors, and additions of elicitors and inhibitors compared to the plant cultivations. Such a bioprocess technology has eased the scale-up process and production improvement; hence, endophytes have emerged as the most explored in vitro platforms of therapeutic secondary metabolites [31]. 

A well-established biosynthetic pathway of the desired metabolite helps design yield enhancement strategies for fungi. Although flavonoid biosynthesis is well understood in plants, the intact biosynthetic pathway in fungi, let alone marine fungi, is still unclear [32,33,34]. 

We hypothesized that our endophyte, *C. globosum,* could be manipulated to enhance chrysin production. We performed metabolic profiling of *C. globosum* extracts to trace the flavonoid biosynthesis pathway. We stimulated *C. globosum* with several biotic and abiotic elicitors, manipulated the culture medium, and observed the trends in the biomass and chrysin production patterns. To the best of our knowledge, this is the first integrated study on chrysin production enhancement from a marine fungus. These findings set the stage for the biotechnological potential of marine endophytes. 

## 2. Materials and Methods

### 2.1. Fungal Strain and Cultivation Conditions

The marine endophytic fungus *C. globosum* PG 1.6 was isolated from a marine algae *Chaetomorpha* sp. collected from the Konkan coast, India, in our previous study [9]. The fungus was maintained on PDA supplemented with 250 mg/mL streptomycin and artificial sea salts. A glycerol stock of ~10^6^ spores/mL was maintained. An inoculum size of ~2 × 10^4^ spores/mL was used for all the experiments by suitably diluting the concentrated spore solution. Any experimental inconsistencies due to an error in the inoculum spore count measurement were accounted for by starting fresh control groups for each study. All the experiments were performed in triplicates, and average values were used for analyses [35]. 

### 2.2. Sample Preparation for MS

To investigate the presence of metabolic intermediates of the flavonoid biosynthesis pathway, *C. globosum* was grown in PDB for 28 days. Further, total culture extraction was performed with four different solvents of varying polarity, viz., methanol, ethyl acetate, and hexane, as described in our earlier work [9]. The extracts were dried and further subjected to LC-MS/MS. 

### 2.3. Metabolite Identification by LC-MS/MS

The samples were analyzed on a Bruker Impact HD ESI-QTOF coupled with a DIONEX Ultimate 3,000 micro-LC system. The source parameters were as follows: Endplate offset 500 V, Capillary 4500 V, Nebulizer 60.0 psi, dry gas 120.0 L/min, dry temperature 220 °C, MS, MS/MS performed in negative and positive mode. Acquisition parameter: Mass scan range 50–1700 *m*/*z*. LC parameters: Column-Agilent poroshell 120 (4.6  ×  150 mm) SB-C18 2.7 µm particle size, column temperature 25 °C. Gradient elution was performed with mobile phases A (water with 0.1% acetic acid) and B (acetonitrile), where B was maintained at 30% from 0 to 0.5 min, 40% from 0.5 to 10 min, 50% from 10–20 min, 70% from 20–30 min, 90% from 30–44.9 min, and 30% from 44.9 to 45 min. 

The MS/MS spectra were analyzed using the Metfrag database (https://msbi.ipb-halle.de/MetFragBeta/ (accessed on 5 November 2022)) [9,36].

### 2.4. Effect of Fermentation Parameters on Fungal Biomass and Chrysin Production in the Suspension Culture of C. globosum

The suspension culture experiments were initiated in 500 mL Erlenmeyer flasks by adding the above-mentioned spore inoculum in 200 mL PDB. It was initially established in our previous study that *C. globosum* grown for 28 days in PDB in static condition demonstrated the highest biomass and cytotoxic activity [9]. Hence, in this study, these conditions served as the control. The effect of the fermentation parameters, namely, media, temperature, and incubation time, was evaluated for the biomass and chrysin production in the suspension culture of *C. globosum* using the single-factor optimization technique. Every parameter was varied within a range while keeping the other parameters the same as the control conditions. 

The effect of different growth media was studied, namely, PDB, M2, MM2, M3, M1D, FBM, MFBM, S7, and MM3, described by Palem et al. [37]. Similarly, we studied the effect of the temperature range 4–35 °C, the incubation time range 7–35 days, and initial pH. The culture was harvested, and the optimum effect of the fermentation parameters was determined to enhance the chrysin concentration. 

### 2.5. Effect of Precursors, Key Intermediates, and Elicitation on Biomass and Chrysin Production in the Suspension Culture of C. globosum

Based on the metabolic profiling obtained from the previous experiment, two key metabolites of the flavonoid biosynthesis pathway, namely, phenylalanine and cinnamic acid, were added separately to the suspension culture of *C. globosum*. Elicitation is one of several biotechnology strategies designed and applied for productivity enhancement. We elicited the fungus with several biotic and abiotic elicitors. The levels of precursors and elicitors used in this study and the corresponding literature are listed in Table 1. The precursor treatment and elicitation were provided on the 14th day of the incubation period. The effect on fungal biomass and chrysin production was evaluated on the 28th day after harvesting the culture.

The optimized fermentation conditions and the levels of precursors and elicitors were used to initiate a combined study. Further, the biomass and chrysin production were evaluated and compared with that of *C. globosum* cultivated in PDB before optimization. 

### 2.6. Analytical Methods

#### 2.6.1. Biomass Estimation

To study the biomass profile, the suspension culture was harvested after the cultivation period of 28 days. The fungal mycelium and culture filtrate were separated by filtration with Whatman No 1 filter paper. The fungal biomass was gently washed in saline and spread on a pre-weighed Whatman No 1 filter paper for overnight drying in a hot air oven. The dry weight of the fungal biomass was determined thereby in g/L.

#### 2.6.2. Extraction and Estimation of Chrysin Production

As reported earlier [9], chrysin was extracted from the total culture extract of *C. globosum.* The dried fungal biomass was crushed and added back into the fermented broth. Extraction was performed for 24 h using twice the volume of the previously optimized organic solvent ethyl acetate. Further, the organic and aqueous phases were separated, after which the organic phase was dried under vacuum, using a rotary evaporator at 40 °C [38] to obtain a concentrated residue. 

The amount of chrysin in the several samples of *C. globosum* ethyl acetate extract was estimated using HPLC. The separation was accomplished on a C18 column using the mobile phases 50% methanol and 50% acetonitrile water at a 1.0 mL/min flow rate. The absorbance of chrysin was measured at 310 nm at the column temperature 30 °C. The retention time of chrysin was 24.49 min. The amount of chrysin in each sample was calculated by making a standard plot of chrysin (97% purity; Sigma-Aldrich, Bengaluru, India). The estimation metrics were chrysin concentration (mg/L) and chrysin yield (mg/g of dry biomass weight) [35]. 

**Table 1:** Strategies used for chrysin optimization.

**Strategy 1: Optimization of Fermentation Parameters**		
Medium optimization	PDB, M2, MM2, M3, M1D, FBM, MFBM, S7, MM3	[37]
Incubation time	7–35 days	[35]
pH	4.0–9.0	[39]
Incubation temperature	4–35 °C	[35]
**Strategy 2: Precursor/Key Biosynthetic Pathway Metabolite Feeding**		
Phenylalanine	0–7 µM	[40]
Cinnamic acid	0–1.5 mM	[40]
**Strategy 3: Elicitation**		
Biotic		
Polysaccharide (Sodium alginate)	0.5–1.5 mg/mL	[41]
Yeast extract	300–800 µg/mL	[42]
Abiotic		
UV radiation	0–15 min	[43]
Salinity stress (NaCl doses)	0–5.5%	[41,44]
Metal stress (CdCl_2_)	0–1 mM	[45,46]

## 3. Results and Discussion

### 3.1. Metabolic Profiling of Chrysin Biosynthesis Intermediates by LC-MS/MS

We detected four metabolites in the methanolic extract and three in the ethyl acetate extract (Table 2). The MetFrag scores of all the hits were close to 1. We attributed the absence of hits in the hexane extract to the polar nature of flavonoids, the extraction of which will not be supported by the non-polar nature of hexane solvent. We used a threshold MetFrag score of 0.9 for the metabolites. We attribute the limited number of intermediates detected in our study to either unknown modifications of flavonoids (sulfated) [47] that are absent in the database or metabolite degradation. 

Since the flavonoid biosynthesis pathway is uncharacterized in fungi, we referred to the pathway proposed by Miyahisa et al. [48] and Zhao et al. [49]. Miyahisa et al. [48] introduced the flavanone 3β-hydroxylase and flavone synthase genes into recombinant *Escherichia coli* and observed the production of several flavones and flavanols, including chrysin. Zhao et al. [49] reported an evolved flavone biosynthetic pathway in the roots of *Scutellaria baicalensis*, a medicinal plant. The specialized flavones produced by this plant were wogonin and baicalein; these lack the 4′-hydroxyl group in the B-ring of the structure. Both flavones could induce apoptosis in several human cancer cells and inhibit tumor progression in vivo. The plant has a flavone synthase II-2 enzyme that is specifically responsible for synthesizing root-specific flavones, such as chrysin and wogonin. Other flavones, such as wogonoside, baicalein, and baicalin, were synthesized from chrysin in the roots. The authors also reported the cinnamic acid-specific coenzyme A ligase and an isoform of chalcone synthase genes, highly expressed in roots for synthesizing chrysin and other flavones in the roots. Modifications, such as methylation, glycosylation, hydroxylation, and prenylation, introduce flavonoid diversity [50]. The key enzymes of this pathway include phenylalanine ammonia-lyase (PAL), cinnamate-4-hydroxylase (C4H), 4-coumarate-CoA ligase (4CL), chalcone synthase (CHS), and chalcone isomerase (CHI). The final product is a flavanone, which is further converted to a series of flavonoids under the catalytic action of different enzymes [51].

However, the flavonoid biosynthesis pathway in fungi has not yet been elucidated. Monhanta [34] reported the presence of key genes of the flavonoid biosynthesis pathway (PAL, CHS, CHI, and flavanol reductase) in the mushroom genome. The editorial cautioned that the presence of these key genes is not universal in fungi. Metabolomics using liquid chromatography with tandem mass spectrometry (LC-MS/MS) aims to detect metabolites in biological samples [52]. Since we reported chrysin from a marine fungus, we intended to investigate the presence of the flavonoid biosynthesis pathway in the marine fungus *C. globosum*. We used the targeted metabolomics approach to identify key metabolites of the pathway and thus get clues about the presence of the flavonoid biosynthesis pathway in a marine fungus.

Our study detected some key intermediates of the flavonoid biosynthetic pathway. In plants, flavonoids have a critical role in assisting the tolerance to abiotic stress, such as salinity, UV-B, and drought stress [53]. Salinity, high temperature, and UV-B exposure are the major abiotic stressors experienced by the host green alga *Chaetomorpha media* and its endophytes. Flavones, flavanols, and isoflavones are routinely detected in algae [54]; thus, the presence of a biosynthetic pathway to flavonoids in algae and its endophytic organisms cannot be ruled out.

### 3.2. Effect of Fermentation Parameters on Biomass and Chrysin Production in the Suspension Culture of C. globosum

Metabolite yield can be enhanced by the optimization of fermentation parameters, such as production media, temperature, and time interval. The classical one-factor-at-a-time (OFAT) method can achieve efficient, economic enhancement of a desired metabolite [55]. 

#### 3.2.1. Quantification of *C. globosum* Biomass and Chrysin in Different Media

**Figure 1:**
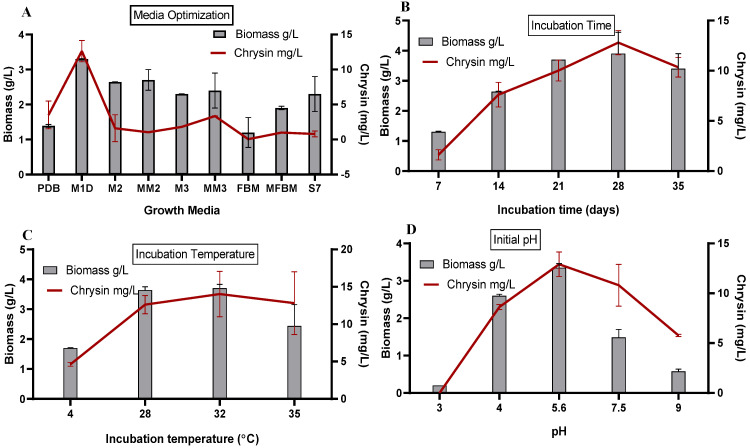
Effect of fermentation parameters on biomass and chrysin production in the suspension culture of *C. globosum*. (**A**) Media optimization; (**B**) Incubation time; (**C**) Incubation temperature; (**D**) Initial pH.

Medium optimization is one of the critical studies performed before any large-scale production process. The fungus *C. globosum* was grown in nine different growth media to establish the optimum condition for producing chrysin using the OFAT strategy. After an incubation period of 28 days, total culture extraction was performed with ethyl acetate, as described earlier [9]. As seen in Figure 1A, the maximum biomass (3.73 g/L) and chrysin concentration (12.62 mg/L) were observed in the fungus grown in M1D medium at pH 5.6, followed by MM2 and MM3 media set at pH 6.0. Sucrose serves as the carbon source in the M1D medium. In a classical study by Walsh and Harley [56] on sugar absorption by resting *C. globosum*, it was reported that the fungus possesses an enzyme system located on the hyphal surface that splits sucrose. Although the fungus preferred glucose over sucrose, the carbohydrate utilization difference accounted for a small value of 4 mg after 18 h at 25 °C in static conditions. In the same study, the fungus supplied with sucrose demonstrated a biomass of 20 mg, while that of the glucose-supplemented fungus was 17 mg. The authors observed that sucrose uptake and its breakdown to hexose depended on normal fungal metabolism. Notably, the Km for sucrose was 2.5 × 10^−4^ and was not significantly different from that of glucose 9 × 10^−4^ [56]. Sucrose is also known to hydrolyze rapidly under acidic conditions. Another classical study reported the active mycelial growth and sucrase activity of *Pyronema conlluens* and *Chaetorniuni cochloides* grown in sucrose-supplemented medium [57]. Our fungus, *C. globosum*, is associated with marine algae. The host alga *Chaetomorpha* sp. is known to synthesize and accumulate sucrose for osmotic adjustment in salt stress [58]. This could also be the reason for the active growth of *C. globosum* in M1D medium containing sucrose. Hence, we chose M1D as the growth medium for all further experiments.

#### 3.2.2. Effect of Incubation Time Interval on C. globosum Biomass and Production of Chrysin

The fungus was grown for 35 days in M1D suspension culture in static conditions (Figure 1B). The biomass and chrysin production gradually increased across the time points. We recorded the maximum biomass of the fungus (3.81 g/L) and chrysin production (12.80 mg/L) in 28-day-old culture. Beyond 28 days, the fungal growth and chrysin production were reduced [9,23]. We have attributed this to decreased carbon sources and other micronutrients in the culture medium and observed that beyond 28 days, the fungus adapts to nutrient-deficient conditions by limiting its growth. Although some studies report increased secondary metabolite production in the stationary phase [23,59], we observed a concomitant relationship between fungal biomass and chrysin production. Our finding agrees with Yahaya and Don (2014), who optimized the incubation time for optimum flavonoid production by the fungus *Trametes lactinea* [60]. Therefore, we chose 28 days incubation time to maximize chrysin production while maintaining sufficient fungus growth. 

#### 3.2.3. Effect of Temperature on C. globosum Biomass and Production of Chrysin

The effect of temperatures in the range 4–35 °C was studied on the biomass and chrysin production of *C. globosum* grown in M1D suspension culture for 28 days in static conditions. We recorded the least biomass and chrysin concentration for fungus grown at 4 °C and the maximum biomass (3.7 g/L) and chrysin concentration (14.02 mg/L) for fungus grown at 32 °C. Hawker and Chaudhari (1946) [57] also observed maximum dry weight and sucrose utilization of *Chaetomium* sp. at 32 °C. The production of chrysin increased with the rise in temperature. However, at 35 °C, the biomass levels dropped, and that could be the reason for the slight drop in chrysin production (Figure 1C). Camptothecin production from the endophyte *Fusarium solani* demonstrated a similar trend [35]. Higher temperatures could favor the activity of enzymes involved in chrysin production. Yahaya and Don (2014) observed the highest flavonoid production at 35 °C, the highest fungal biomass at 30 °C, and a substantial decrease in biomass above 30 °C. The authors maintained 35 °C to maximize flavonoid production [60]. To ensure maximum chrysin production and sufficient fungus growth, we maintained *C.globosum* at 32 ± 2 °C for subsequent studies.

#### 3.2.4. Effect of Initial pH on C. globosum Biomass and Production of Chrysin

The effect of initial pH over the 3.0–9.0 range was studied on the biomass and chrysin production of *C. globosum* grown in M1D suspension culture for 28 days at 32 ± 2 °C in static conditions (Figure 1D). We recorded the highest biomass of 3.35 g/L at an initial pH of 5.6 and the least biomass at pH 3.0 and 9.0. We found the highest production of chrysin, 12.91 mg/L, at an initial pH of 5.6, followed by pH 7.0, although the biomass level reduced. Haida et al. [39] reported the highest biomass and flavonoid production within the initial pH range from 5.0 to 6.0 for the *Ficus deltoidea* var. *kunstleri*. Several studies have documented the retardation in cell growth and flavonoid production outside this pH range [39]. Extreme pH, i.e., below 4.0 and above 7.0, can affect growth. A mild acidic environment induces acidification in the cytoplasm and triggers the expression of the phenylalanine ammonia lyase gene, a key enzyme involved in flavonoid biosynthesis [61]. To ensure maximum chrysin production while maintaining sufficient fungus growth, we maintained *C. globosum* at pH 5.6 for subsequent studies.

### 3.3. Effect of Addition of Precursor and Key Intermediate on C. globosum Biomass and Production of Chrysin

**Figure 2:**
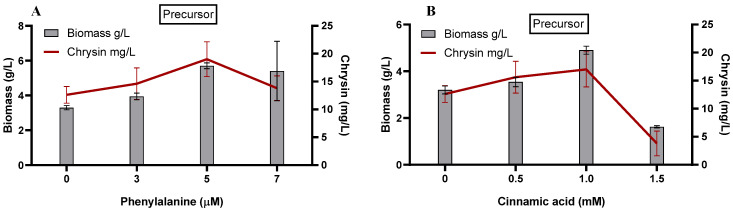
Effect of precursor feeding on *C. globosum* biomass and production of chrysin. Flavonoid biosynthesis pathway precursor (**A**) phenylalanine and key intermediate (**B**) cinnamic acid.

The precursor of chrysin biosynthesis, phenylalanine, and a key intermediate, cinnamic acid, were added at specific concentrations to the *C. globosum* suspension culture under optimized conditions on day 14 of the incubation period (Figure 2). Phenylalanine is the precursor of the flavonoid biosynthesis pathway that is converted to cinnamic acid by the catalytic action of phenylalanine ammonia lyase (PAL). Cinnamic acid is converted to cinnamoyl-CoA by the 4-coumaroyl–CoA ligase (4CL)-like enzyme [49,62]. In our study, the addition of 5 µM phenylalanine (Figure 2A) increased the biomass production up to 5.7 g/L, compared with the control. Chrysin production also accelerated gradually, resulting in the highest concentration of 19 mg/L at 5 µM phenylalanine. A further increase in the phenylalanine dose resulted in a deacceleration of the chrysin production, although the biomass production remained relatively constant.

Interestingly, *Aspergillus oryzae,* when grown in a minimum medium containing L-phenylalanine as a sole source of nitrogen, converts L-phenylalanine to 2-phenylethanol, which limits the biomass growth [63]. We infer that *C. globosum* utilizes yeast extract (nitrogen source) in M1D while utilizing phenylalanine for flavonoid production. However, after the nitrogen source in M1D is exhausted, phenylalanine is utilized as the nitrogen source that produces 2-phenylethanol. 

Cinnamic acid addition (0–1.5 µM) resulted in maximum biomass (4.90 g/L) and chrysin production (17.02 mg/L) at 1 µM cinnamic acid elicitation (Figure 2B). At 1.5 µM cinnamic acid addition, the biomass level and chrysin production demonstrated a sharp decrease of 1.62 g/L and 3.80 mg/L, respectively. Cinnamic acid is a key metabolite that regulates several metabolic pathways in microbes. In plants, it acts as an allelochemical that influences seed germination and root growth. It induces ROS production, thus causing oxidative stress that increases flavonoid production. However, cinnamic acid at a higher concentration diverts the phenylpropanoid pathway toward lignin biosynthesis [40]. Therefore, we anticipate an increase in other secondary metabolites that restrict the growth of *C. globosum*. 

### 3.4. Effect of Elicitation on C. globosum Biomass and Production of Chrysin

The elicitation strategy improves the production of desirable secondary metabolites sustainably in a limited time. Exogenous elicitors are compounds or a condition that impart stress and promote secondary metabolism; this may result in higher production or new structures of specific secondary metabolites. Specific receptors and ion channels in the plasma membrane perceive the elicitors, causing a change in gene expression to mitigate the stress [64]. Elicitation can be biotic (polysaccharides, yeast extract, enzymes, bacteria, fungi) or abiotic (high salinity, ultraviolet radiation, heavy metals, oxidative stress, drought) [65]. 

Flavonoids respond to environmental or developmental stimuli, and they are known for their antioxidative and shielding properties against UV radiation and their salinity resistance, thus conferring a stress defense [43,66]. In this study, we elicited *C. globosum* with biotic and abiotic elicitors to trigger the maximum production of chrysin. 

#### 3.4.1. Effect of Biotic Elicitation

##### Polysaccharide: Sodium Alginate (NaAlg)

**Figure 3:**
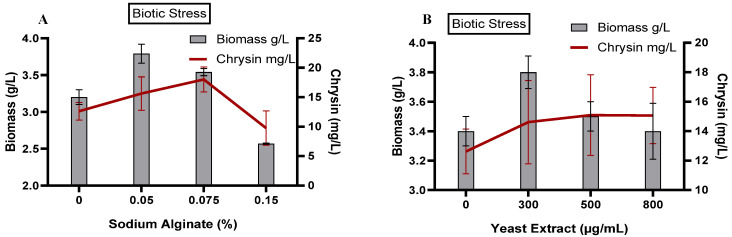
Effect of biotic elicitors on *C. globosum* biomass and production of chrysin. (**A**) Sodium alginate (NaAlg); (**B**) Yeast extract (YE).

NaAlg, a natural polysaccharide extracted from brown algae (Phaeophyceae), is a potential elicitor for the biosynthesis of various secondary metabolites. A significant increase in chrysin production was observed in the presence of NaAlg (Figure 3A). The highest biomass was recorded at 0.05% NaAlg, while the highest chrysin production was at 0.075% NaAlg. At 0.15% NaAlg, both biomass and chrysin production decreased. Several studies have recorded increased biosynthesis of secondary metabolites, such as morphine, codeine, artemisinin, and flavonoids, when elicited with NaAlg [44,67]. Ryder et al. (1984) observed a significant increase in the expression of the chalcone synthase gene—a regulatory enzyme of the flavonoid biosynthesis pathway—in *Phaseolus vulgaris* when elicited with polysaccharides of the *Colletotrichin lindemuthianum* cell wall [68]. NaAlg and chitosan elicitation of *Vitis vinifera* L. cv. Cabernet Sauvignon cell suspension cultures significantly elevated the production and accumulation of stilbene and trans-resveratrol while maintaining the biomass [69]. Polysaccharides and oligosaccharides of plant or fungal cell walls trigger defense responses and the production of defense-related metabolites [70,71]. In our study, we have anticipated the production of other defense-related metabolites, along with chrysin, all of which could contribute to a reduction in biomass levels after exposure to more than 0.15% NaAlg. 

##### Yeast Extract (YE)

Elicitors of fungal origin, such as yeast extract, chitosan, and chitin, have been routinely used in stimulating the production of various secondary metabolites, such as alkaloids, flavonoids, coumarin derivatives, and terpenoids [72]. The highest biomass production of 3.8 g/L was recorded at 300 µg/mL YE, after which the biomass production slightly reduced to 3.5 g/L and 3.4 g/L (Figure 3B). Chrysin production increased from ~12 mg/L in the control; to ~15 mg/L, when the fungus was supplemented with with 300 µg/mL YE; beyond which the biomass and chrysin production reduced. Goyal and Ramawat reported an increased isoflavonoid accumulation in cell cultures of *Pueraria tuberosa* supplemented with YE [42]. YE elicitation also stimulated the production of stilbenes, rosmarinic acid, artemisinin, podophyllotoxin, paclitaxel, and lignin [73]. Zhao et al. (2012) treated sprout cultures of *Fagopyrum tataricum* with yeast polysaccharide and observed a significant increase in phenylalanine ammonia lyase (PAL), a key entrance enzyme of the flavonoid biosynthesis pathway, coupled with flavonoid accumulation and, interestingly, sprout growth [74]. 

#### 3.4.2. Effect of Abiotic Elicitation

##### UV Radiation

**Figure 4:**
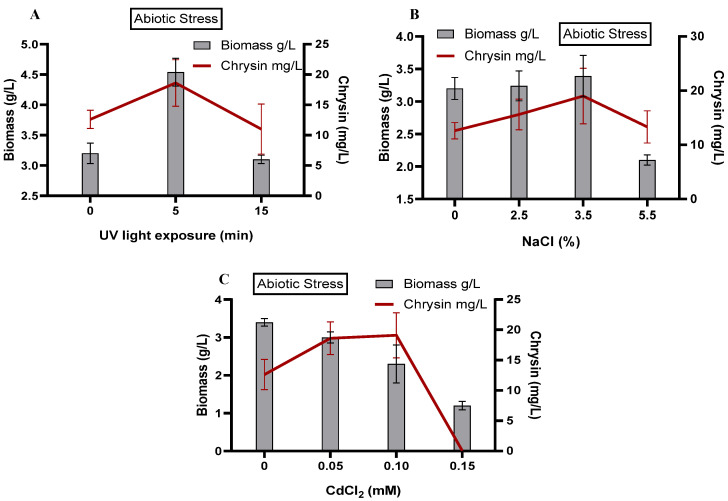
Effect of abiotic elicitors on *C. globosum* biomass and production of chrysin. (**A**) UV radiation exposure; (**B**) Salinity stress (NaCl doses); (**C**) Metal stress (CdCl_2_).

In this study, we elicited *C. globosum* with non-ionizing radiation: UV radiation stress for 5 min and 15 min. UV radiation of 5 min resulted in maximum chrysin production of 18.61 mg/L and biomass of 4.54 g/L. A further increase in the UV radiation duration reduced the biomass and chrysin production (Figure 4A). El-Bialy and El-Bastawisy [75] used UV radiation as a mutagenesis strategy to elicit paclitaxel production in three endophytes: *Acremonium*, *Colletotrichum*, and *Fusarium* spp. Out of the three endophytes, elicited *Acremonium* produced fourfold more paclitaxel than its unstimulated counterpart due to the increased activity of a key gene of the paclitaxel biosynthesis pathway. However, the normal fungal morphology and growth declined with an increasing UV dose. 

##### Salinity Stress (NaCl)

We demonstrated the effect of varying NaCl or salinity stress doses on *C. globosum* biomass and the production of chrysin (Figure 4B). A 3.5% NaCl elicitation resulted in a maximum biomass of 3.39 g/L and chrysin production of 19.02 mg/L. A further increase in NaCl elicitation drastically reduced the biomass to 2.0 g/L, which was less than the unelicited group; the chrysin production was similar to the unelicited group. The effects of salinity stress are osmotic stress and altered ionic balance, such as the K^+^/Na^+^ ratios. A combination of these effects causes fungi to produce ROS, resulting in oxidative damage and altered growth and metabolism [76]. To mitigate salt stress, a salt-tolerant plant endophyte, *Chaetomium globosum* D5, Ref. [77] accumulated soluble proteins and metabolites, namely, polyphenolics, dismutase, and catalase. Salinity stress in safflower plants triggered a 36% increase in PAL enzyme activity and, thus, an increase in the total flavonoid and phenolics content [41]. Salinity stress induces water loss, membrane disruption, metabolic imbalance, and excessive accumulation of ROS, thus leading to oxidative damage [66]. Samec et al. (2021) studied the effect of salinity stress in *Brassica* leafy vegetables; high salinity doses elicited a significant increase in polyphenolics and a significant decrease in carotenoids. Interestingly, salt stress also accumulated sodium in the vegetables, adding an undesirable value to otherwise healthy vegetables. Therefore, the authors caution against using salinity elicitation in vegetables [76]. These observations progressively support the role of endophytic fungi in the growth and survival of marine alga. 

##### Heavy Metal Stress (CdCl_2_)

Heavy metal stress enhances the production of secondary metabolites, such as resveratrol from *Vitis vinifera* [45] and tanshinones from *Salvia miltiorrhiza* cultures [78]. As seen in Figure 4C, biomass production was reduced with an increase in the CdCl_2_ elicitor treatment. The production of chrysin was highest (~19 mg/L) for 0.05 and 0.1 mM elicitation. However, at 1.5 mM elicitation, the fungal growth and chrysin production sharply decreased. Overall, the results indicate the strong stimulatory effect of Cd^2+^ on fungal growth, which also affects chrysin production. Bilal et al. observed the protective effect of the endophyte *Penicillium* sp. on metal-induced stress in plants. Although the metal stress impeded the fungal growth, the protective effect was due to the elevated levels of expression of flavonoid biosynthesis genes [46]. Heavy metal stress induces an oxidative burst, quickly activating catalases, peroxidases, and flavonoid biosynthesis pathways. Hussain et al. (2022) reported the presence of a heavy-metal-tolerant endophyte, *Aspergillus welwitschiae,* that could improve the survival of the host plant soybean [79]; the fungus showed high flavonoid content and enzymatic antioxidants. 

Compared with the precursor feeding study, *C. globosum* elicited with abiotic stressors demonstrated lower biomass. Elicitation demonstrated a positive correlation with flavonoid production up to a specific range [66,80], also observed in our study. 

### 3.5. Verification of the Fermentation Parameters, Precursors, and Elicitors

A combined study was performed based on the optimal values of the various fermentation parameters, precursor and intermediate feeding, and elicitors. *C. globosum* was grown in suspension culture using M1D medium for 28 days at 32 °C and supplemented with precursors, a biotic, and biotic elicitors (described in Table 3) on the 14th day. The combined influence of all the parameters caused a dramatic increase in chrysin and biomass production. A *C. globosum* biomass of 4.05 g/L could produce a chrysin concentration of 21.02 mg/L. Thus, the yield of chrysin after optimization of the parameters was 5.18 mg/g, compared with 2.53 mg/g before optimization, when the fungus was grown in PDB, a 97-fold increase. Thus, we developed a fungal cell factory that could produce chrysin. 

## 4. Conclusions

The results obtained in this work demonstrated the valuable potential of metabolomics and bioprocess optimization strategies. This work can serve as a template for the enhanced production of a desired secondary metabolite from a new source. We have also established the significance of exogenous supplementation of precursors and elicitors for yield enhancement of a secondary metabolite from a new source. Further, elicitation can be investigated using other abiotic and biotic factors, such as salicylic acid, methyl jasmonate, glucose, and co-culture with fungi, plant cells, and bacteria. Statistical tools, such as the response surface methodology, can be applied to enhance the productivity manifoldly. Marine endophytic fungi live in a robust and stressful environment, making them attractive sources of biologically active compounds. The unique conditions, such as pH, temperature, salinity, and nutrient availability, and the symbiotic relationship with higher organisms, such as algae, corals, and sponges, contribute significant chemical diversity in the fungal metabolome. In this study, we explored the robust nature of a marine endophytic fungus, *C. globosum*, to enhance the production of chrysin, a bioactive flavone, and achieve higher production of chrysin by manipulating the media and culture conditions and the biotic and abiotic elicitation of *C. globosum*. This work underscores a sustainable route to enhance the production of desired compounds using endophytic fungi. 

## Figures and Tables

**Table 2:** Metabolites of the chrysin biosynthesis pathway detected in the methanolic, ethyl acetate, and hexane extract of *C. globosum* by LC-MS/MS.

RT (min)	Observed Mass *m*/*z*	Theoretical Mass *m*/*z*	Intermediate	MetFrag Score
**Methanolic Extract**				
8.2	288.06538	288.063	Dihydrokaempferol	0.97
24.3	254.258	254.241	Chrysin	1
24.8	270.253	270.24	Galangin	0.95
34.1	270.053	270.24	Baicalein	0.90
**Ethyl Acetate Extract**				
1.9	913.152	913.61	p-Coumaroyl-CoA	1
18.2	897.157	897.70	p-Cinnamoyl-CoA	1
2.2	324.159	324.4	Isobavachalcone	0.91
**Hexane Extract [Nil]**				

**Table 3:** Verification of optimized fermentation conditions, precursors, and elicitors for enhanced chrysin production.

Parameter	Variable
Media	M1D
Incubation time	28 days
Incubation temperature	32 °C
Phenylalanine	5 µM
Cinnamic acid	1 mM
YE	300 µg/mL
NaAlg	0.075%
UV light exposure	5 min
NaCl	3.5%
CdCl_2_	0.05 mM
Biomass	4.05 g/L
Chrysin concentration	21.02 mg/L
Chrysin yield	5.18 mg/g
Initial chrysin yield	2.53 mg/g

## Data Availability

The data that support the findings of this study are available from the corresponding author upon reasonable request.

## Supplementary Materials

The following supporting information can be downloaded at: https://www.mdpi.com/article/10.3390/microorganisms11040999/s1, Figure S1: Chrysin from marine endophytic fungus.
