# Supplementary material for: Improved Chrysin Production by a Combination of Fermentation Factors and Elicitation from Chaetomium globosum
Source: Microorganisms. 2023 Apr 12;11(4):999. doi: 10.3390/microorganisms11040999 (PMC10146793; doi:10.3390/microorganisms11040999)
Supplement: Supplementary file 1 [file microorganisms-11-00999-s001.zip › microorganisms-2275873-supplementary.pdf]

Improved Chrysin Production by a Combination of Fermentation Factors and Elicitation from *Chaetomium globosum*

Siya Kamat[1], Madhuree Kumari[1], Kuttuvan Valappil Sajna[1,2], Sandeep Kumar Singh[3], Kaushalendra[4], Ajay Kumar*[5] and C. Jayabaskaran*[1]

1. Department of Biochemistry, Indian Institute of Science, Bangalore, 560012, India, siyakamat@iisc.ac.in (S.K.); madhuree88@gmail.com (M.K.); cjb@iisc.ac.in (C.J.)
2. Celignis Biomass Analysis Laboratory, Limerick V94 7Y42, Ireland. sajna@celignis.com.
3. Division of Microbiology, Indian Agricultural Research Institute, Pusa, New Delhi 110012, India; sandeepksingh015@gmail.com
4. Department of Zoology, Mizoram University (A Central University), Pachhunga University College Campus, Aizawl-796001, India; kaushalpuc@gmail.com
5. Centre of Advanced Study in Botany, Banaras Hindu University, Varanasi-221005, India; ajayku mar_bhu@yahoo.com

Correspondence: ajaykumar_bhu@yahoo.com (A.K.) and cjb@iisc.ac.in (C.J.)

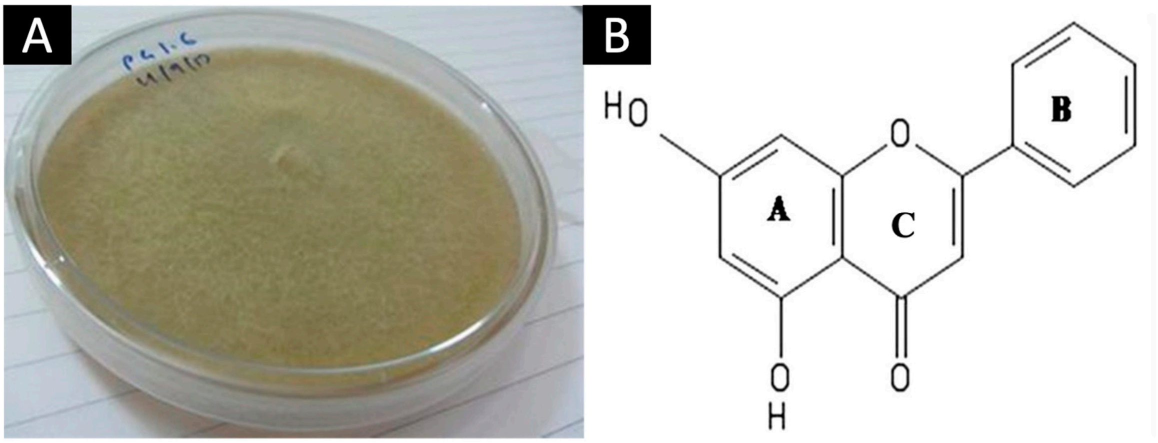

Supplementary Figure 1: Chrysin from marine endophytic fungus (A) *Chaetomium globosum* growing on Potato Dextrose agar (B) Structure of chrysin with classic A, B and C rings that make the flavones.
